# Explainable semi-supervised deep learning shows that dementia is associated with small, avocado-shaped clocks with irregularly placed hands

**DOI:** 10.1038/s41598-023-34518-9

**Published:** 2023-05-06

**Authors:** Sabyasachi Bandyopadhyay, Jack Wittmayer, David J. Libon, Patrick Tighe, Catherine Price, Parisa Rashidi

**Affiliations:** 1 J. Crayton Pruitt Family Department of Biomedical Engineering, University of Florida, Gainesville, USA; 2 Department of Computer and Information Science and Engineering, University of Florida, Gainesville, USA; 3 Department of Geriatrics and Gerontology, Department of Psychology, New Jersey Institute for Successful Aging, School of Osteopathic Medicine, Rowan University, Glassboro, USA; 4 Department of Anesthesiology, College of Medicine, University of Florida, Gainesville, USA; 5 Department of Clinical and Health Psychology, College of Public Health and Health Professions, University of Florida, Gainesville, USA

**Keywords:** Machine learning, Diagnostic markers, Biomedical engineering, Computational science, Diagnostic markers, Psychology, Dementia, Dementia

## Abstract

The clock drawing test is a simple and inexpensive method to screen for cognitive frailties, including dementia. In this study, we used the relevance factor variational autoencoder (RF-VAE), a deep generative neural network, to represent digitized clock drawings from multiple institutions using an optimal number of disentangled latent factors. The model identified unique constructional features of clock drawings in a completely unsupervised manner. These factors were examined by domain experts to be novel and not extensively examined in prior research. The features were informative, as they distinguished dementia from non-dementia patients with an area under receiver operating characteristic (AUC) of 0.86 singly, and 0.96 when combined with participants’ demographics. The correlation network of the features depicted the “*typical dementia clock*” as having a small size, a non-circular or *“avocado-like”* shape, and incorrectly placed hands. In summary, we report a RF-VAE network whose latent space encoded novel constructional features of clocks that classify dementia from non-dementia patients with high performance.

## Introduction

**Figure 1:**
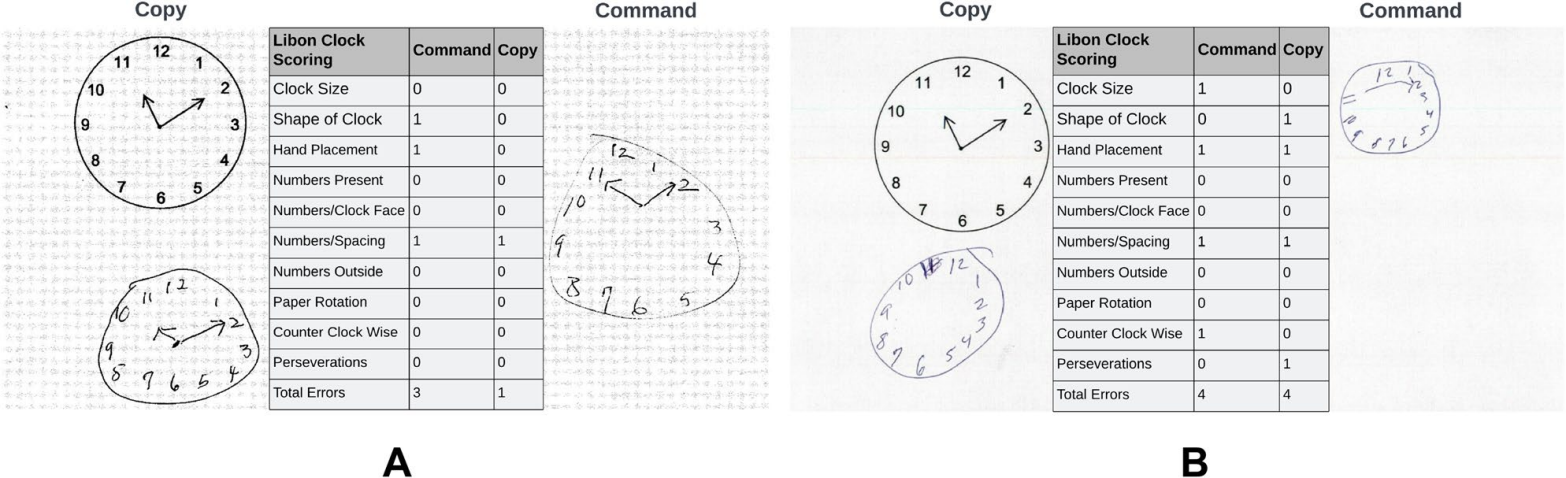
Example clock drawing tests with Libon scoring. (**A**) Clock drawing to command and copy conditions by the first individual. Clocks were scored on various metrics by the Libon criteria. This scoring system adjudicated the command clock to have atypical shape, hand placement and numbers’ spacing. It also adjudicated the copy clock to have erroneous number spacing. (**B**) Clock drawing to command and copy conditions by the second individual. The Libon criteria decided that the command clock has atypical size, hand placement, numbers’ spacing, and is drawn in counter-clockwise direction. The copy clock was evaluated to have anomalous shape, hand placement, numbers’ spacing and to have digit repetitions (perseveration).

Clock drawing is a simple, effective, and inexpensive way to screen for cognitive impairment in individuals with suspected mild cognitive impairment (MCI) or dementia, including Alzheimer's disease (AD) and vascular dementia (VaD). The CDT consists of two parts: the command test condition, where participants are required to “draw the face of a clock, put in all the numbers, and set the hands to *ten after eleven*”; followed by the copy test condition where participants are instructed to copy a model clock. Two example clock drawings are shown in Fig. 1 with their corresponding annotations using Libon scoring criteria^1^. Accurate clock drawing depends on the coordination of a host of cognitive abilities. Subtle changes in clock drawing behavior can reveal intricate details about underlying cognitive functioning^2,3^. Command condition drawing requires the ability to process linguistic components of verbal instructions, syntactic comprehension of these instructions, recalling the semantic attributes of a clock, working memory, effective mental planning, visuospatial processing, and motor skills to execute the drawing effectively^4^. Drawing the copy condition clock requires visual scanning ability, visuocontruction, and executive functioning to complete the task^5,6^. Command and copy condition drawings have been shown to test complementary cognitive abilities^7^. Also, performance on the CDT is correlated with other alternative assessments of cognitive frailty, e.g., the Mini-Mental State Examination (MMSE)^7,8^.

Previous literature has explored various ways of analyzing the CDT, ranging from nominal (good/bad) to elaborate 22 or 31-point analog scoring systems^2,9–11^. These systems have tried to describe the CDT based on salient features in the clock drawing where subtle changes can indicate the onset of cognitive ailments. Some scoring systems have been based on analysis of errors assessing semantics, graphomotor functioning, and executive control^2^. Despite having similar psychometric properties^12^, these scoring protocols hinge on the examiner’s ability to interpret a participant's output leading to potentially unreliable results^13^. For example, Price and colleagues have found considerable variance in intra and inter-rater reliability^14,15^. The human component required for interpreting the CDT can also introduce ambiguities that can potentially reduce the robustness of any diagnosis. The THink project attempted to eliminate this variability by codifying all analysis routines which partook in scoring^16^. They also introduced the digital clock drawing test (dCDT)^16^ to analyze the temporal component of the CDT. The dCDT uses a digital pen and smart paper technology to capture the temporal order of all pen strokes in patients’ drawings. Using dCDT technology, multiple novel clock drawing elements such as latencies between pen strokes, the total number of pen strokes, and total time taken to execute the drawing are made available for analysis^17^. Davis et al.^16^ used approximately 500 spatio-temporal features from the dCDT to classify Dementia versus Healthy with accuracy = 0.82, AUC = 0.70, F1 = 0.46, Alzheimer's versus Healthy with accuracy = 0.84, AUC = 0.76, F1 = 0.69, using linear support vector machines (SVM).

Despite the vast number of features extracted by the dCDT, they are eventually handcrafted by domain experts. Handcrafted features cannot span the entire space of relevancy and may suffer from redundancy. Therefore, Binaco et al.^18^ extracted 350 dCDT features to maximize joint and conditional mutual information and minimize redundancy. They achieved AD versus non-MCI accuracy = 0.91, amnestic MCI versus non-MCI accuracy = 0.83, mixed/dysexecutive MCI versus non-MCI accuracy = 0.85, all MCI versus non-MCI accuracy = 0.84 over a tenfold cross-validation using feed-forward neural network classifiers^18^. Davoudi et al.^19^ further streamlined the features from Binaco et al.^18^ into 37 kinematic, time-based, and visuospatial features. They used this set of features to classify a combined group of AD and VaD from healthy controls with AUC = 0.91, Accuracy = 0.91, Specificity = 0.97, Sensitivity = 0.71, F1-score = 0.80 using random forest classifier^19^. These methods have used machine learning and information-theoretic measures to extract informative and non-redundant features from the dCDT.

Alternatively, deep learning (DL) can automatically extract a nested hierarchy of features of increasing complexity using backpropagation of errors. Several studies have used deep convolutional neural networks (CNN) for scoring CDTs^20,21^. Some studies have used CNN variants (for e.g., R-CNN, U-Net) for segmenting a clock drawing into its individual components (clockface, numbers and hands) and used additional CNN models to score them separately^22,23^. DL models such as CNN typically comprise millions of trainable parameters requiring commensurately large, labeled datasets to train them effectively. Otherwise, they converge to local suboptimal states which are not generalized or robust, thus limiting their clinical utility. These models are time and resource-intensive, requiring the collection and annotation of large datasets to train them from scratch. In the absence of large labeled datasets, traditional supervised DL models cannot extract objectively important features. To circumvent this problem, researchers have used CNN models pre-trained on large datasets such as MNIST or ImageNet which have no bearing on clock drawings. This approach significantly hinders model interpretability and the DL system is merely used as a “black-box” predictor. In contrast, in this study we have used a deep, generative, semi-supervised DL model to create an interpretable predictor.

Recent advances in Artificial Intelligence have provided us with alternative methods to extract features that are (1) informative, (2) disentangled and (3) complete^24^ in an unsupervised way. This paper uses a state-of-the-art deep generative model named relevance factor variational autoencoder (RF-VAE) to capture all meaningful observable sources of variation in the clock drawing in an unsupervised way^25^. RF-VAE is an advancement on the variational autoencoder (VAE), a generative model that learns a joint probability distribution over all variables present in a dataset in an unsupervised manner^26^. RF-VAE leverages the latent space's total correlation (TC) to achieve the disentanglement goal. It focuses the TC loss onto the relevant factors by tolerating a large prior Kullback–Leibler (KL) divergence while simultaneously eliminating nuisance factors of variation with small prior KL divergences^25^. It uses a suite of disentanglement metrics to demonstrate that RF-VAE outperforms existing methods across several challenging benchmark datasets^25^.

The primary aim of this project is to calibrate clock drawing construction using a focused set of informative, disentangled constructional features that are useful for discriminating dementia from non-dementia peers. The study is formulated as a semi-supervised learning task where a large unlabeled dataset of clock drawings was used to train the RF-VAE network in an outcome-agnostic way. The trained model encoder was then fine-tuned together with a feed-forward, fully-connected neural network to classify dementia from control participants. Hyperparameters, including the number of relevant latent dimensions in the RF-VAE network, were optimized based on the classification performance. The RF-VAE decomposes the clock drawing into an optimal number of independent latent features linked to specific aspects of clock construction. The feed-forward neural network classifier combines these features in a non-linear way to discriminate dementia from controls. A previous study attempted to classify dementia from non-dementia using a two-dimensional latent space VAE network^27^. This work provided proof of concept that compressed CDT representations retain their ability to distinguish dementia. Our results expand on this fundamental preliminary finding by cataloguing a complete set of independent and informative graphomotor features of clock drawing which can distinguish dementia from controls with high performance. To the best of our knowledge, these results represent a pioneering step in developing explainable semi-supervised deep learning models using CDT for identifying dementia.

## Results

### Participants

This study is a multi-center, multi-cohort study performed in collaboration between the University of Florida and the Rowan University, New Jersey. Three cohorts were used in this study namely-training cohort, fine-tuning cohort and testing cohort. Table 1 shows the participants' demographics in the training and classification (fine-tuning and testing) cohorts. All participants in the classification cohort completed both command and copy condition drawings. Three individuals in the training cohort could not complete the command condition. In the classification cohort, dementia participants were significantly older, had lower MMSE scores, and had fewer years of education than their non-dementia peers. The training cohort had an equal percentage of male and female participants, whereas the classification cohort was predominantly male. Furthermore, there were significantly more male individuals in the dementia cohort. Both the training and classification cohorts had a predominance of white people.

**Table 1:** Demographics of cohorts.

Dataset	Number of samples	Mean age (S.D)	Mean education (S.D)	% of female	% of caucasian	Mean MMSE total score	Mean MoCA total score
Training clocks	23,521	73 (6)	14 (3)	50	86	26 (4.0)	N/A
Dementia	112	80 (6)	13 (3)	32	98	22 (2.6)	N/A
Controls	350	68 (6)	16 (2)	46	95	29 (0.9)*	25 (2.3)^†^

*S.D* standard deviation, *MMSE* mini-mental state examination, *MoCA* Montreal cognitive assessment.

*This value is available only for 126 participants in the controls cohort.

^†^This value is available only for 224 participants in the control cohort.

### RF-VAE latent space (training dataset)

**Figure 2:**
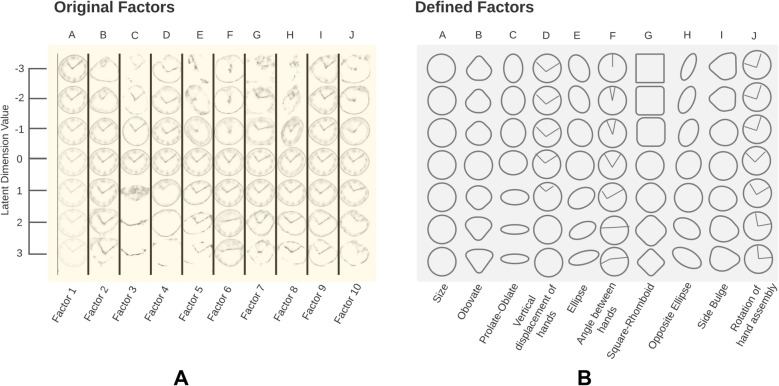
Description of the latent space learned by the RF-VAE in the training phase. (**A**) Reconstructed clock drawings are shown as a function of every latent variable. Each column represents a single latent dimension ranging from − 3 (top) to + 3 (bottom). (**B**) A simplified explanation of every latent space traversal is provided. Each latent dimension is described by a constructional aspect of the clock drawing, which most closely resembles the traversal of reconstructed clocks over this particular latent dimension.

Figure 2A shows the RF-VAE trained latent space after completion of unsupervised training with 23,521 clock drawings from both command and copy conditions. Each column corresponds to one latent dimension, and represents traversal over the latent space along that dimension. Due to disentanglement, there was no cross-correlation between these latent dimensions in the training dataset (Supplementary Fig. 1). Figure 2B defines the nature of each latent variable and elucidates its change over the corresponding latent dimension.

Column A shows a change in the brightness of the clock drawing. In reality, this corresponds to the size of the clock drawing as clocks of various sizes were resized to 64 × 64 during preprocessing, resulting in a decrease in the brightness of the larger clocks. Column B shows the existence of ovate and obovate (avocado-shaped) clocks in the training dataset. The direction of orientation of the obovate clock reverses as this latent dimension increases. This increase is associated with a lengthening of the clock hands. Column C encodes the change of clock shape from prolate (elongated) to oblate (flattened) with an increase in its latent dimension. Column D shows an upward movement of the point of intersection of the clock hands from the geometric clock center, with an increase in its latent dimension. Column E shows the presence of eccentric ellipsoidal clock drawings. The direction of the eccentricity of ellipsoidal clocks changes from left to right as this latent dimension increases. Column F shows an increase in the angle between the clock hands as its latent dimension increases. Column G shows the existence of non-circular clocks in the dataset. An increase in this latent dimension changes the clock shape from square to circular to rhomboid. Column H again shows ellipsoidal clocks, but in this case, the orientation changes from right to left as the latent dimension increases. Therefore, this dimension is the logical opposite of the fifth latent dimension. Column I shows the presence of clocks that have a horizontal circular asymmetry (side bulge). The side bulge changes position from left to right as the latent dimension increases. Column J shows a rotation of the clock hands while maintaining a constant inter-hand angle. This indicates clocks where the subject put hands in numbers other than 11 and 2 or a general shift in the placement of digits in the clock. These are the ten disentangled constructive imperfections identified by the RF-VAE network from the training dataset. In the case of all factors, a shift towards higher absolute value of the latent variable is associated with the loss of digits on the clockface (Fig. 2A).

### RF-VAE latent space (classification dataset)

**Figure 3:**
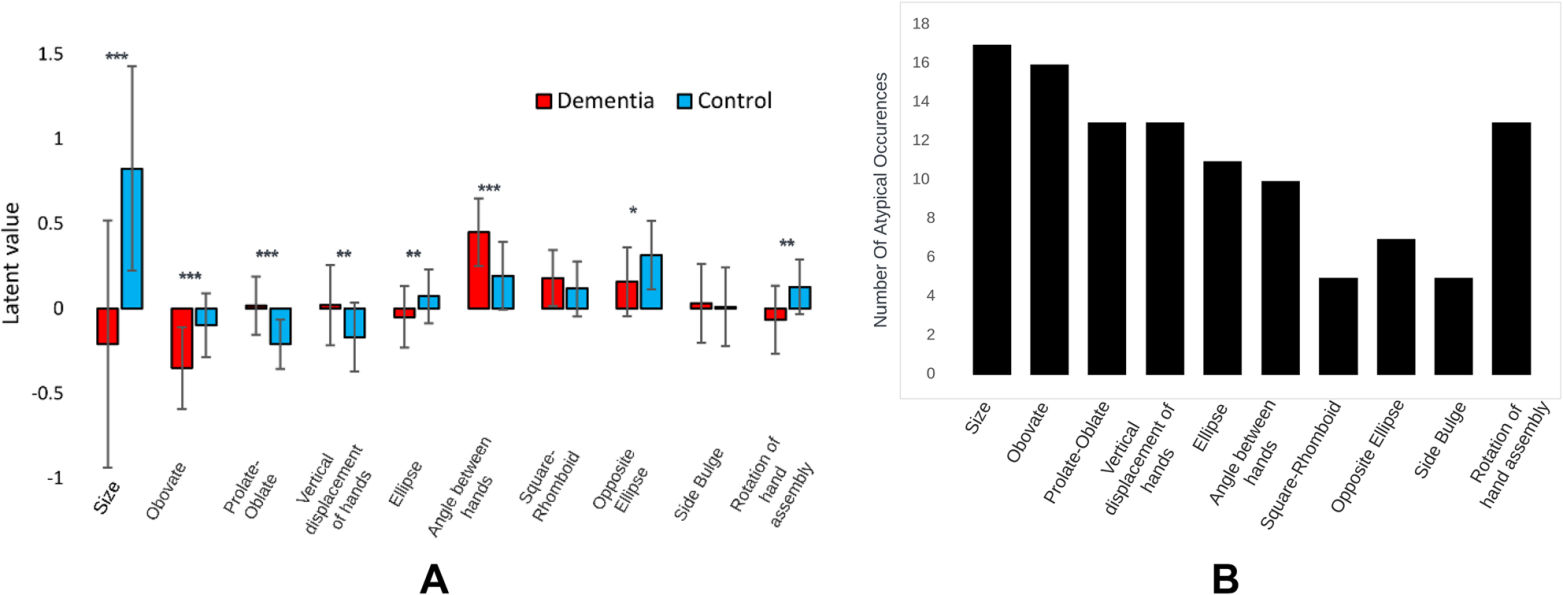
Statistical comparison between dementia and non-dementia samples over the ten latent variables. (**A**) Mean and Standard Deviation of each latent variable in the dementia and the non-dementia group. ***p < 0.0001, **p < 0.001, *p < 0.01. *p* values were generated after propensity matching to remove confounding effects of age and education and after multiple comparisons correction using FDR = 0.01 on two-tailed Student’s T-tests (Supplementary Table 1). (**B**) Number of “atypical occurrences” of latent variables in the dementia group. These atypical occurrences were calculated by the number of times a latent variable value in a dementia clock was greater than two standard deviations from the mean of the control distribution.

All clocks in the classification dataset contained these anomalies to different degrees. Supplementary Figs. 2A–I show the distribution of each feature among dementia and non-dementia participants. Figure 3A shows the comparison between mean and standard deviations of each feature between dementia and non-dementia groups after removing confounding effects of age and education through propensity matching. Significance was inferred from *p* values calculated after multiple comparisons correction on two-tailed, unequal variance Student’s T-test using the Benjamini–Hochberg method (False Discovery Rate; FDR = 0.01). Uncompensated *p* values are provided in Supplementary Table 1 for reference. Clock size shows the greatest difference between dementia and non-dementia distributions. Features attributed to the clock shape such as obovateness, prolate/oblateness, ellipticity and those attributed to clock hands such as vertical displacement, angle between hands and rotation of hands show significant difference between dementia and non-dementia groups. Comparing the latent values in Fig. 3A to Fig. 2A shows that dementia clocks are considerably smaller, obovate, oblate clocks with vertically displaced hands having large angle between them. Rotation of the clock hand assembly showed the maximum drop in significance after compensating for age and education differences (Supplementary Table 1). Square-rhomboid and side-bulge have bi-modal dementia distributions and unimodal non-dementia distributions (Supplementary Fig. 2G,I) although they are not significantly different between dementia and non-dementia groups. Furthermore, we found the number of “atypical occurrences” of each feature in the dementia group by comparing them against the mean and standard deviation of the respective non-dementia distribution (Fig. 3B). Size has the highest number of atypical occurrences in the dementia cohort. Square-rhomboid and side-bulge have the least number of atypical occurrences in the dementia cohort. Size, obovateness, prolate-oblateness, vertical displacement of clock hand assembly, and rotation of clock hand assembly are most frequently atypical in dementia clocks.

**Figure 4:**
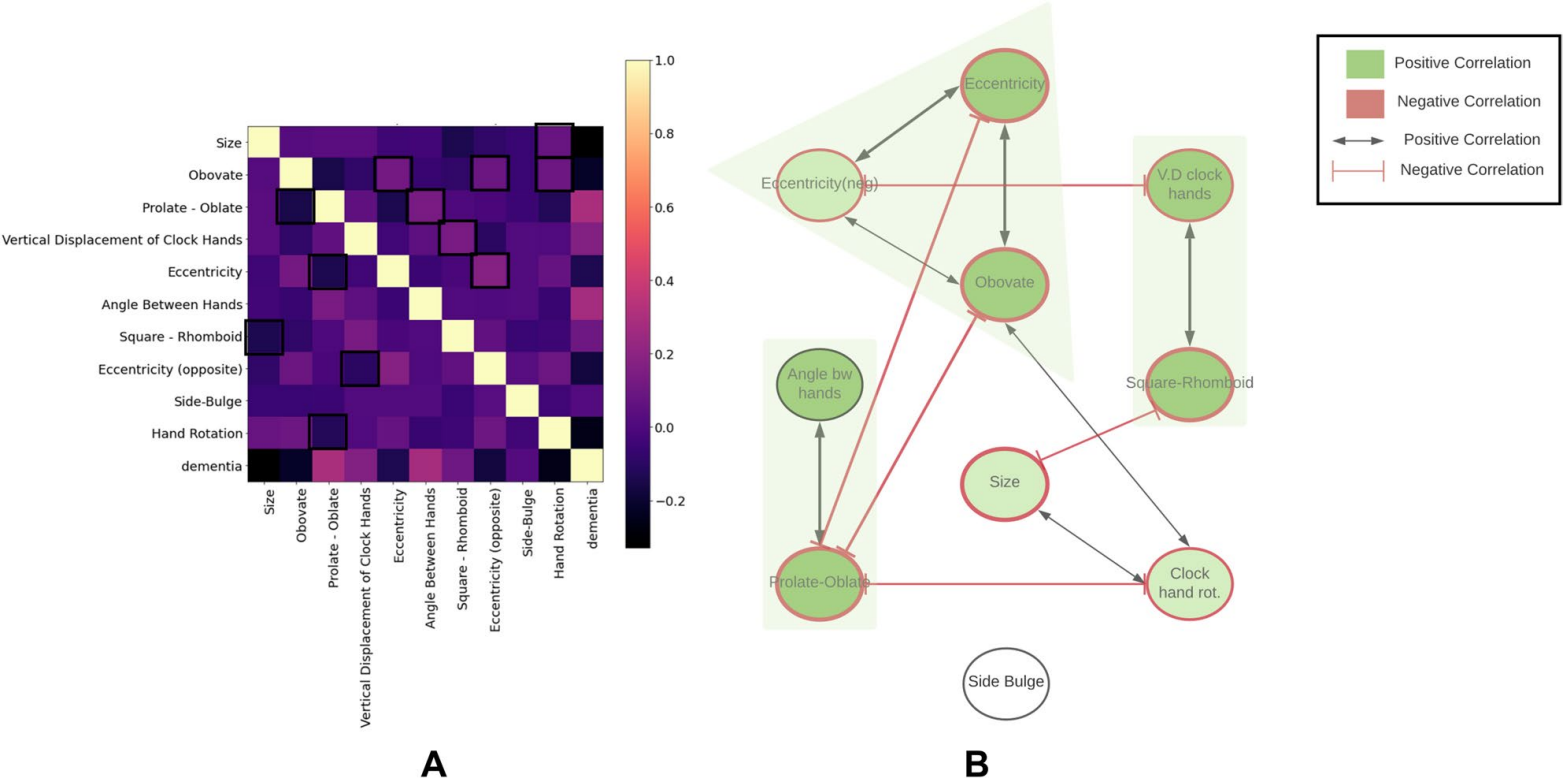
Correlation patterns of latent RF-VAE features in the classification dataset. (**A**) Cross-correlation matrix between the ten latent features found by the RF-VAE. Correlation between each latent dimension and dementia label is also shown. (**B**) Feature co-occurrence network was constructed using only the relatively high correlation values as the adjacency matrix. Green denotes a positive correlation. Red denotes a negative correlation. Black arrows with regular arrowheads denote positive correlation, and red arrows denote negative correlation. The width of the arrows denotes the relative strength of correlation. Three subnetworks emerge where intra-subnetwork features are positively correlated with one another, and inter-subnetwork features are negatively correlated with each other. Clock size and clockwise rotation of clock hand assembly are weakly correlated. Side-bulge is not significantly correlated to any other feature.

We examined the cross-correlation between different latent variables on the classification dataset and found the presence of positive and negative correlations (Fig. 4A). We used these correlations as adjacency values of a graph to represent the relations between the latent variables in a graphical format (Fig. 4B). The graph depicts the presence of three subnetworks characterized by relatively high intra-network positive correlation (correlation > 0.2) and inter-network negative correlations (correlation < − 0.2). The three subnetworks comprise (a) obovate—eccentricity, (b) vertical displacement of clock hands—square/rhomboid, and (c) prolate/oblate—angle between clock hands. Prolate/oblate is negatively correlated with eccentricity and obovate. Vertical displacement of clock hands is negatively correlated with eccentricity. Furthermore, clock size and rotation angle of clock hand assembly show a weak positive correlation (correlation ~ 0.1). Clock size is negatively correlated with square/rhomboid. Clock hand rotation is negatively correlated with prolate/oblate. Finally, the dementia label is correlated with small clock size, avocado-shape, flattening of the clock face (oblateness), eccentricity, increasing angle between hands, and anticlockwise rotation of the hand assembly.

### Classification performance (fine-tuning and testing datasets)

We simultaneously fine-tuned the weights of the RF-VAE encoder and trained a neural network classifier with the fine-tuning dataset. The ten latent variables generated by the RF-VAE encoder were input to the classifier firstly as standalone features and secondly with demographics (age, sex, race, and years of education) for distinguishing dementia from non-dementia. The test dataset was used to report the final performance metrics on both occasions, as shown in Table 2. 95% confidence intervals show the robustness of the model's performance over bootstrapped versions of the test data. The model achieves good performance on the test data simply using the ten latent variables and achieves almost perfect classification when demographics are added to the model. The classification performance using solely demographic information is presented for reference.

**Table 2:** Performance of classifier on test data.

Task	Features	AUC (95% C.I.)	Accuracy (95% C.I.)	F1- score (95% C.I.)	Precision (95% C.I.)	Sensitivity (95% C.I.)	Specificity (95% C.I.)	NPV (95% C.I.)
Dementia prediction	10 latent variables	0.86 (0.79–0.93)	0.84 (0.77–0.91)	0.72 (0.59–0.84)	0.62 (0.44–0.76)	0.9 (0.80–1.0)	0.82 (0.73–0.89)	0.96 (0.91–1.0)
Dementia prediction	10 latent variables + demographics	0.96** (0.89–0.99)	0.95** (0.90–0.99)	0.88** (0.77–0.96)	0.82** (0.66–0.94)	0.96* (0.85–1.0)	0.95** (0.90–0.99)	0.99** (0.97–1.0)
Dementia prediction	Demographics	0.81 (0.73–0.88)	0.86 (0.80–0.93)	0.72 (0.63–0.80)	0.58 (0.49–0.67)	0.70 (0.61–0.78)	0.92 (0.87–0.97)	0.95 (0.88–1.0)

*AUC* area under the curve, *C.I* confidence interval, *NPV* negative predictive value.

*p value < 0.01.

**p value < 0.001.

## Discussion

RF-VAE delineated ten constructional features in clocks drawn by participants as part of a routine medical assessment in a preoperative setting. The ten constructional factors are as follows (1) size, (2) degree and orientation of obovate, (3) prolate–oblate, (4) vertical displacement of the point of intersection of clock hands, (5) degree and direction of ellipticity, (6) angle between clock hands, (7) square—rhomboid clockfaces, (8) degree and direction of ellipticity in an opposite sense than (5), (9) degree and direction of side-bulge of clockface, and (10) rotation of clock hands assembly.

**Figure 5:**
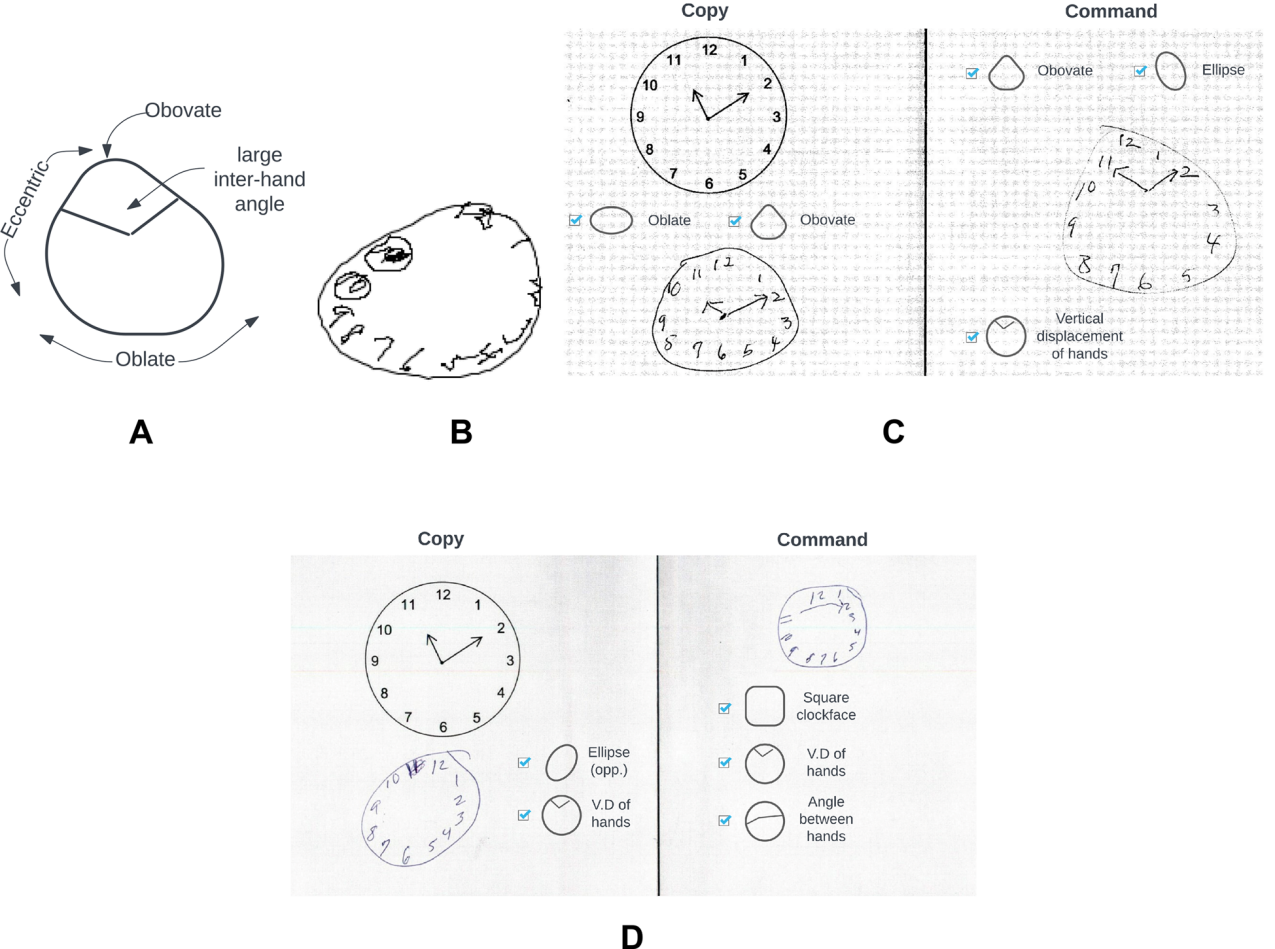
Combination of atypical values of features that are significantly associated with dementia. (**A**) Our model found that dementia clocks are small, avocado shaped (obovate dimension < 0), oblate (prolate/oblate dimension > 0), eccentric (ellipse dimension < 0), have large inter-hand angle, and hands rotated in an anticlockwise direction. (**B**) This clock drawing was given the highest likelihood of being dementia by our model. (**C**) Command and copy clocks drawn by the first individual with errors discovered by the latent variables of RF-VAE (to be compared against Fig. 1A). (**D**) Command and copy clocks drawn by the second individual with errors discovered by the latent variables of RF-VAE (to be compared against Fig. 1B).

These factors are deemed independent generative factors that are significant sources of variation in clock drawings by the unsupervised training of a RF-VAE. Each clock comprised a superposition of these factors to different degrees. Statistical comparison of the different latent features between dementia and non-dementia showed that in our dataset dementia was most typically associated with small, avocado-shaped, oblate clocks with irregularly placed hands. Figure 5A shows a hypothetical clock drawing comprising a combination of the latent variables most highly associated with dementia in our dataset. Figure 5B shows the clock which was given the highest probability of being dementia by our neural network classifier.

These latent variables could distinguish dementia from nondementia peers with superlative performance, and the addition of age, sex, race, and years of education resulted in the near-perfect classification of dementia from non-dementia in the test dataset. The model's high performance using standalone latent variables as features proves that these features are highly informative of the participants' cognitive status. However, significant improvement upon the addition of demographics proves that demographics still contain non-redundant information necessary for the classification of dementia from controls.

The factors discovered in this study are generally different from traditional analog metrics used to score a clock drawing test, such as digit placement accuracy, missing digits, hand placement accuracy and the ratio of hour hand to minute hand length. The RF-VAE latent variables generally describe a global change in the shape of the clockface and placement of clock hands, whereas dCDT features describe salient high resolution graphomotor and latency variables from the CDT. Despite broad differences, some similarities exist. The ratio between the lengths of major and minor axes in a clock drawing is reflected in the fifth and eighth latent dimensions (degree and direction of the eccentricity of the ellipsoid) of RF-VAE. Similarly, hand misplacement corresponds to latent dimensions four (vertical displacement of the point of meeting of clock hands), six (angle between clock hands), and ten (rotation of clock hand assembly from 11 and 2). Figure 5C,D show which factors are atypically expressed in the CDTs shown in Fig. 1. By comparing Fig. 1A,B with Fig. 5C,D we can appreciate that the RF-VAE factors represent the graphomotor elements of a clock drawing in a novel and more nuanced way than traditional scoring criteria.

Despite disentanglement being a requirement in discovering these features, some features are algorithmically associated. For example, a more oblate clock will have greater angle between clock hands, and a change in the shape of the clock face from circular to square can vertically displace the clock hand assembly. These relations are reflected in the three subnetworks found from the classification dataset's cross-correlation patterns between variables. These data show that the statistical disentanglement achieved by RF-VAE does not necessarily translate to algorithmic independence between the features. Despite statistical disentaglement, the presence of algorithmic dependence between different constructional aspects of the clock drawing can result in correlations between variables in smaller datasets such as our classification cohort. Achieving algorithmic independence between generative features is a possible future course of research in this area. Finally, the weak positive correlation between size and clockwise rotation of the clock hand assembly defines the ideal clockface.

Some of these factors have been identified by domain experts as important in classifying different subtypes of dementia and other cognitive frailties. For instance, a smaller clockface area is associated with subcortical disease profiles with primary executive dysfunction (e.g., micrographia in Parkinson's disease)^9^, and misplacement of clock hands is associated with visual attention deficits and disinhibition^2^. In comparison to a previously published VAE encoding^27^, the RF-VAE encoding reported in this study achieved significantly better results on the same classification dataset using identical training methods. This improvement is due to diversification of the latent space, and disentangling the latent dimensions. Enlarging the latent space allowed us to encode more sources of variations, while disentagling them ensured minimal mutual information.

This project advances bidirectional translational neuroscience with AI. Here, we have used the final result of dCDT to develop and validate a RF-VAE model for identifying dementia in a forward-translational experiment. Clinicians and domain experts can review the disentangled factors identified by the RF-VAE latent space in concert with their classification performance to understand novel feature combinations from the CDT and incorporate them in gold-standard cognitive assessments. This bidirectional opportunity allows domain experts to broaden their understanding of classic cognitive assessments while simultaneously driving the research in futuristic AI technologies with their invaluable domain expertise. This symbiotic association of domain expertise with progressive AI technologies is crucial for fields sensitive to domain-level concerns such as interpretability and mechanistic grounding.

This study has certain limitations. Firstly, the classification performance improvement observed due to demographic features may be traced to the differences in average age and education level between dementia and non-dementia groups. However, this is in line with previous literature that have shown that higher age and lower education increase the risk of dementia in older adults^28–30^. Secondly, the preprocessing step involved resizing all clock images irrespective of their initial size to 64 × 64. This resulted in invariable obfuscation of key clock features such as the shape of digits and the presence of ticks and arrowheads, which can explain their absence from the trained RF-VAE latent space. Furthermore, although the RF-VAE has achieved statistical disentanglement between the latent dimensions, the presence of correlations in the classification dataset points to algorithmic dependence between at least some of these features. Finally, the classification task of separating dementia from non-dementia is considerably general and might not be able to leverage the richness of features identified in the RF-VAE latent space.

In summary, this study showed that factorized VAEs could compress a CDT into a set of highly informative, statistically disentangled latent dimensions. These latent dimensions serve as generative features of the CDT and possess key information on characterizing dementia. We trained the RF-VAE in a completely unsupervised manner and agnostic to any cognitive outcome so that it can identify general, robust features that are informative to any downstream classification task. Thus, the same latent space can be fine-tuned to any downstream classification task related to clock drawings. Due to this advantage inherent in semi-supervised learning, in the future, we aim to represent different cognitive stressors (e.g., surgery, trauma) with a unique combination of the latent variables described here. This will also enable us to better understand and predict the prognosis of cognitive ailments through the CDT. Furthermore, we plan to use the reported RF-VAE latent space to distinguish different types of dementia such as AD, VaD, mild cognitive impairment (MCI), amnestic-MCI, dysexecutive-MCI, and Parkinson's disease. Since our model relies only on the outcome of the CDT it can leverage large amounts of publicly available CDT data for enriching the performance of its disease-specific classifiers.

## Conclusion

In conclusion, in this study we have identified a complete and mutually independent set of graphomotor anomalies which are meaningful sources of variation in the CDT. We have constructed neural network classifiers using these graphomotor features with and without the assistance of participant demographics. Our models were cross-validated for optimal performance and tested on an independent testing cohort to achieve superlative performance in distinguishing dementia from non-dementia clock drawings. In the future, we will expand this study to include post-surgical cognitive dysfunction, Parkinson’s disease and specific types of dementia. We will also use independent publicly available datasets to further validate the features found in this study. This study is a pioneering work in generative feature learning using semi-supervised deep neural networks on clock drawing data.

## Methods

### Participants

Study materials were collected from digital clock drawing consortium data between the University of Florida (UF) and New Jersey Institute for Successful Aging (NJISA), Memory Assessment Program, School of Osteopathic Medicine, Rowan University. The Institutional Review Boards of the University of Florida and Rowan University approved the study. Study participants at both institutions gave their written approval to be included in the study through informed consent forms. All study procedures were carried out per the Declaration of Helsinki and respective university guidelines and TRIPOD criteria^31^. The study consisted of two data cohorts:

*Training dataset* included a set of 23,521 clock drawings from 11,762 participants aged ≥ 65 years, primary English speaking, who completed clock drawing to command and copy conditions as part of routine medical care assessment in a preoperative setting^32^. Exclusion criteria were as follows: non-fluent in the English language; education < 4 years; visual, hearing, or motor extremity limitation that potentially inhibits the production of a valid clock drawing.

*Classification dataset* consists of a “fine-tuning” dataset and a “testing” dataset used to fine-tune and test dementia versus non-dementia neural network classifier, respectively. These datasets comprise clock drawings from individuals diagnosed with dementia and non-dementia peers. The dementia clocks were collected from 56 participants evaluated through a community memory assessment program within Rowan University. They were seen by a neuropsychologist, a psychiatrist, and a social worker. Inclusion criteria: age ≥ 55. Exclusion criteria: head trauma, heart disease, or other major medical illness that can induce encephalopathy; major psychiatric disorders; documented learning disability; seizure disorder or other major neurological disorder; less than 6th-grade education, and history of substance abuse. All individuals with dementia were assessed using the Mini-Mental State Examination (MMSE), serum studies and an MRI scan of the brain. These individuals have been described in previous studies^33^. As reported in previous studies, they were either diagnosed with AD or VaD using standard diagnostic criteria^34,35^.

A total of 175 non-dementia participants completed a research protocol consisting of neuropsychological measures and neuroimaging. Two neuropsychologists reviewed all data. Inclusion criteria: age ≥ 60, English primary language, availability of intact activities of daily living (ADLs) as per Lawton and Brody's Activity of Daily Living Scale, completed by both the participant and their caregiver^36^. Exclusion criteria: clinical evidence of major neurocognitive disorder at baseline, as per the Diagnostic and Statistical Manual of Mental Disorders—Fifth Edition^37^, presence of a significant chronic medical condition, major psychiatric disorder, history of head trauma/neurodegenerative disease, documented learning disorder, epilepsy or other significant neurological illness, less than 6th grade education, substance abuse in the past year, major cardiac disease, and chronic medical illness-induced encephalopathy. These participants were screened for dementia over the telephone using the Telephone Interview for Cognitive Status (TICS^38^) and one in-person interview with a neuropsychologist and a research coordinator who also evaluated comorbidity rating^39^, anxiety, depression, ADLs, neuropsychological functioning, and digital clock drawing^40^. Data from these participants have been described in other studies^3,19^.

### Procedure

Cohort participants completed two clock drawings: (a) command condition where they were instructed to “Draw the face of a clock, put in all the numbers, and set the hands to *ten after eleven*”, and (b) the copy condition wherein the participant was presented with a model of a clock and asked to copy the same underneath it^2^. A digital pen from Anoto, Inc. and associated smart paper^17^ were used to complete the drawings. The digital pen captures and measures pen positions on the smart paper 75 times/second. 8.5 × 11 inch smart paper was folded in half, giving participants a drawing area of 8.5 × 5.5 inch. Only the final drawing was extracted and used for analyses in the current study.

Clock drawings to both command and copy conditions from the training cohort were used to train the RF-VAE. After that, clock drawings to both command and copy conditions from the fine-tuning cohort were used to train the weights of a neural network classifier and fine-tune the weights of the RF-VAE encoder to distinguish dementia from control clocks. Command and copy clocks were not separated in training because we wanted the model to learn clock encodings that are agnostic to any cognitive outcome and hence generalizable to multiple different classification tasks. The fine-tuning dataset comprised 84 dementia and 263 nondementia clocks. Ultimately, the classification network was tested on the test dataset comprising 28 dementia and 87 control clocks.

Individual clock drawings were extracted from the file using contour detection. The extracted contours were cropped to the boundaries of the clock drawing, padded with white space to a square, and resized to 64 × 64, as this was the only size supported by the RF-VAE implementation^25^ used. Supplementary Fig. 3 shows the preprocessing pipeline described above.

### Statistical testing

The latent features developed by the RF-VAE were tested for statistical difference between dementia and non-dementia cohorts using two-tailed Student’s T-tests with multiple comparisons correction using the Benjamini–Hochberg method^41^ with FDR = 0.01. The confounding effects of age and education were removed using propensity score matching using the open-source Python library called PsmPy^42^. This gave us a propensity-score matched cohort of 110 dementia clocks and 220 non-dementia clocks. Significance shown in Fig. 3A were based on adjusted p-values estimated on this propensity-matched cohort, as shown in Supplementary Table 1. Correlation between the variables was calculated using Pearson’s Product Moment Correlation coefficient. Thereafter, the correlation matrix was thresholded at 0.2 and − 0.2 as these values represented 5th and 95th percentiles in the non-parametric distribution of the correlation values. The thresholded binary matrix was used as an adjacency matrix to generate a cross-correlation graph between the latent variables.

### Models and experimental setup

A variational autoencoder (VAE) represents a generative model that can learn a lower-dimensional representation of input data in the form of the mean and standard deviation of a Gaussian distribution which it samples to reconstruct the input data. The non-linear output decoder network compensates the loss of generality caused by the prior normal distribution. One disadvantage of the VAE latent distribution is a lack of disentanglement of factors: each latent variable being exclusively responsible for the variation of a unique aspect of the input data. In this paper, we have used an existing implementation of a VAE-based deep autoencoder model that can learn all meaningful sources of variations in clock drawings in its disentangled latent representation. This model, called RF-VAE, uses total correlation (TC) in the latent space to improve disentanglement of relevant sources of variation while tolerating significant KL divergences from nuisance prior distributions while simultaneously identifying factors having low divergence from these nuisance priors as “nuisance sources of variation”. This way, it can learn “all meaningful sources of variations” in its latent space.

**Figure 6:**
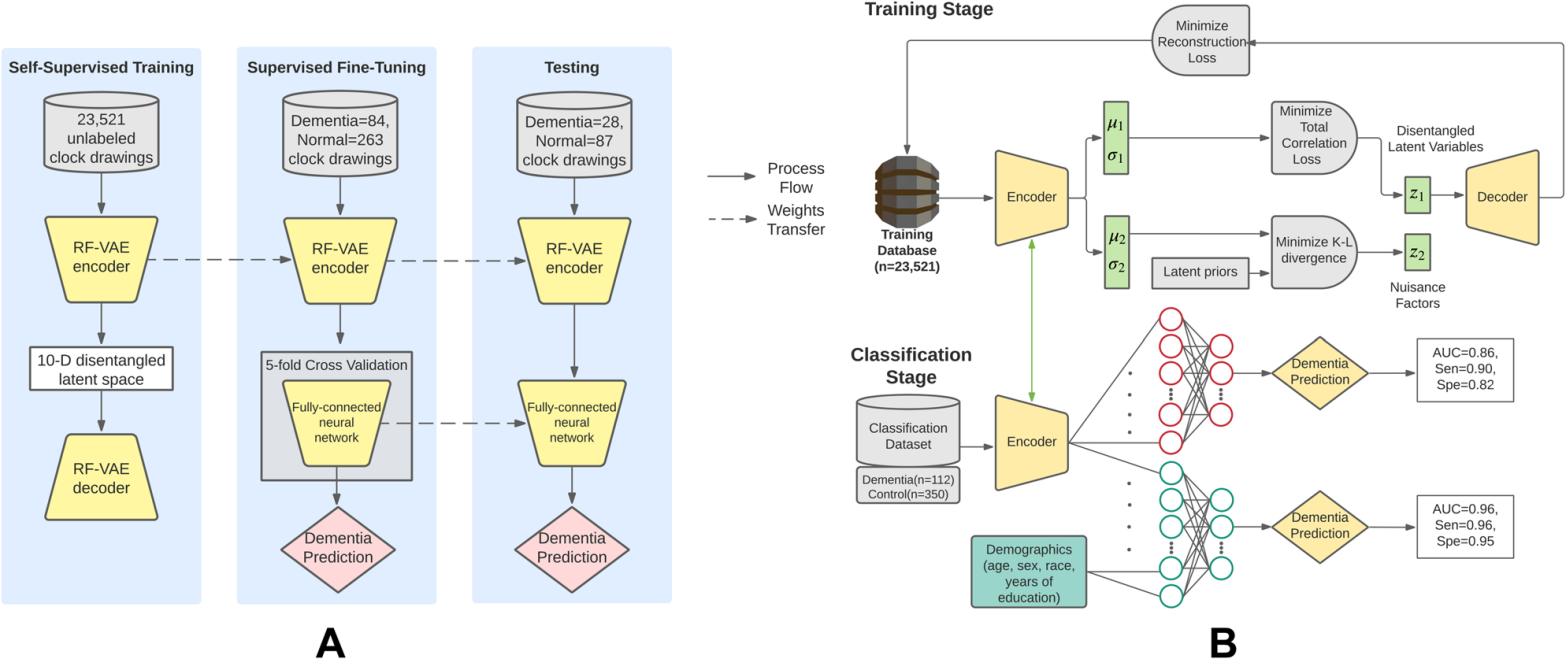
Conceptual workflows of the proposed method. (**A**) High-level conceptual diagram showing the training, validation and testing procedures. RF-VAE undergoes unsupervised training with 23,521 unlabeled clock drawings. Subsequently the trained RF-VAE encoder is transferred to a “fine-tuning” stage where a fully connected neural network is optimized using 84 dementia and 263 normal clocks. Finally, the pre-trained encoder and the fine-tuned classifier are tested on 28 dementia and 87 normal clocks. (**B**) Detailed workflow showing the different loss functions which are minimized during training and classification. In the training stage, a ten-dimensional RF-VAE latent space is constructed by minimizing the loss between original and reconstructed clock drawings and minimizing the total correlation between latent dimensions to disentangle them. Furthermore, feature relevance is ensured in the latent space by eliminating those latent variables that do not diverge significantly from previously defined prior distributions. In the classification stage, the trained encoder is fine-tuned jointly with a fully connected neural network for classifying dementia from non-dementia clocks in the classification stage. Furthermore, age, sex, race, and years of education are added to the latent dimensions to train another classifier with higher performance.

The preprocessed clock image was fed to the RF-VAE network with the latent dimension of 10. The RF-VAE network was trained for 1400 epochs at a learning rate of 10^−4^ with a batch size of 64 following recommendations in source articles^25,43^. The reconstruction loss was cross-entropy, and the optimizer was Adam^44^. RF-VAE training took 3.5 h, on a GeForce GP102 Titan × GPU from NVIDIA Corporation. The trained latent space of the RF-VAE was fed to a fully connected feed-forward neural network with two hidden layers having seven neurons in the first hidden layer and four neurons in the second hidden layer. Using an Adam optimizer, the classifier was trained using the fine-tuning dataset for 20 epochs, with a batch size of 32 and a learning rate of 0.0075. The classification loss was binary cross-entropy. A 3.125:1 weight was assigned to the dementia class during training to ameliorate the class imbalance in the fine-tuning dataset. All hyper-parameters were selected using the fine-tuning dataset inside a fivefold cross-validation design by maximizing the average fold AUC of the model. Figure 6 shows the network architecture and represents our method's conceptual workflow. The top portion of each panel in the figure shows the training process of the RF-VAE. The bottom portion of the figure shows how the trained encoder weights of the RF-VAE support a task-specific classifier. The performance of this trained classifier was tested on the test data, and several important performance metrics, namely, AUC, Accuracy, Sensitivity, Specificity, Precision, and Negative Predictive Value (NPV), were reported. The test data were bootstrapped 100 times using random sampling with replacement to create confidence intervals. The median score, 2.5th quartile, and 97.5th quartile of these metrics over the bootstrapped test dataset were reported.

We evaluated the performance gain of the classifier upon the addition of age, sex, race, and years of education of participants to the model. The best-performing classifier consisted of three hidden layers with ten input neurons, 512 neurons in the first hidden layer, 256 neurons in the second hidden layer, and 128 neurons in the third hidden layer. It was trained for 20 epochs over the fine-tuning data with a batch size of 8, at a learning rate of 0.0075. All hyper-parameters were selected using the fine-tuning dataset inside a fivefold cross-validation design by maximizing the average fold AUC of the model. Figure 6 illustrates the different steps in the workflow.

## Supplementary Information

Supplementary Information.

## Data Availability

Datasets are available upon reasonable request. All dataset related queries should be directed to Dr. Catherine Price (cep23@PHHP.UFL.EDU). Reasonable requests will be reviewed to monitor compliance with the concerned authorities- National Institute of Health (NIH) and the Institutional Review Board (IRB). Relevant clinical trial numbers for the studies from which the datasets in this study have been constructed are NCT01986577 and NCT03175302.

## Code availability

All code used for dataset cleaning, model training, and analysis, as well as the trained RF-VAE model used to encode the clock drawings are provided. They are available at github.com/iheallab/Clock-Drawing-Classification-With-RF_VAE.git.
