# Supplementary material for: Explainable semi-supervised deep learning shows that dementia is associated with small, avocado-shaped clocks with irregularly placed hands
Source: Sci Rep. 2023 May 6;13:7384. doi: 10.1038/s41598-023-34518-9 (PMC10164161; doi:10.1038/s41598-023-34518-9)
Supplement: Supplementary file 1 — Supplementary Information. [file 41598_2023_34518_MOESM1_ESM.docx]

**Supplementary Information**

Explainable Semi-supervised Deep Learning shows that Dementia is associated with small, avocado-shaped clocks with irregularly placed hands

Sabyasachi Bandyopadhyay, MS, Jack Wittmayer, David J. Libon, PhD, Patrick Tighe, MD, MS, Catherine Price, PhD, Parisa Rashidi, PhD.

This supplementary material has been provided by the authors to give the readers additional information about their work.

**Supplementary Figures**


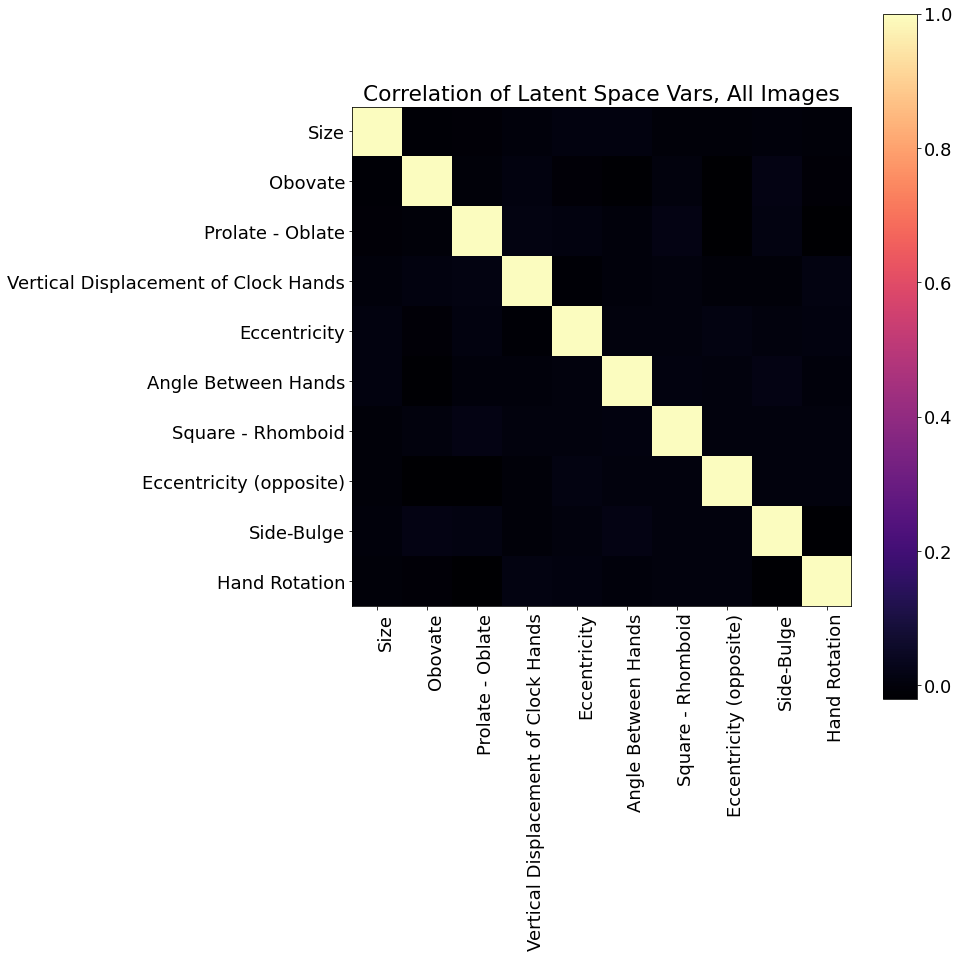


**Supplementary Figure S1. Correlation matrix of latent variables in training dataset.** This figure shows absence of correlation between latent variables in the training dataset, signifying that successful disentanglement has been achieved.


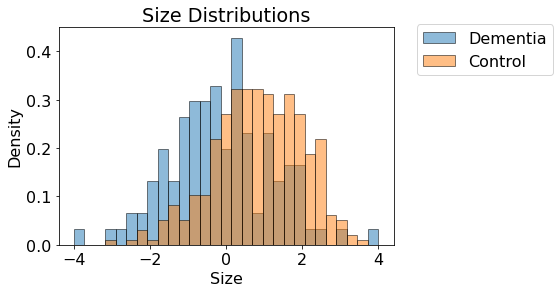

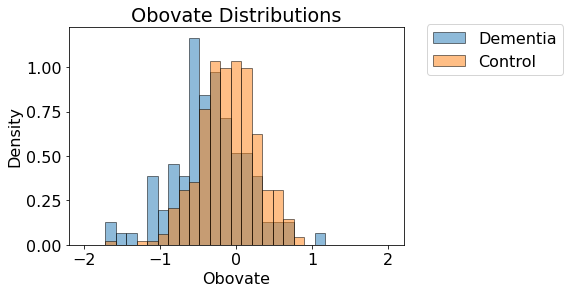


A B


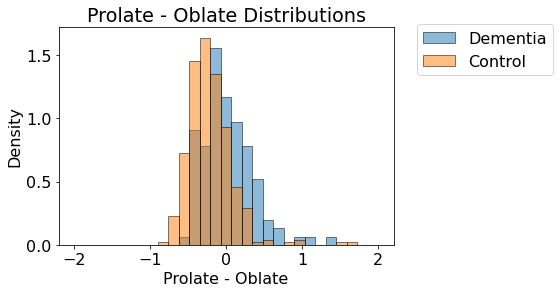

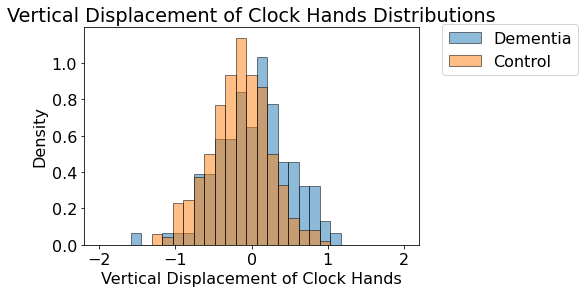


C D


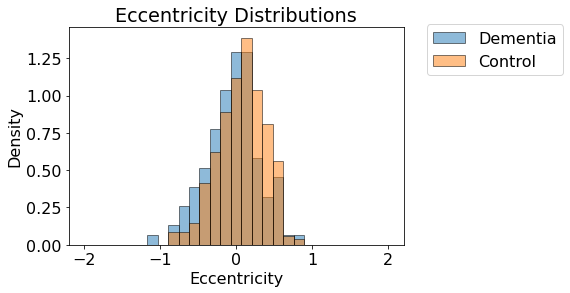

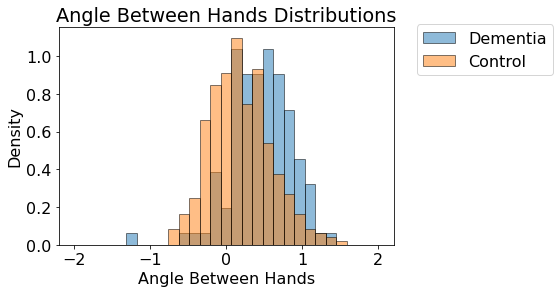


E F


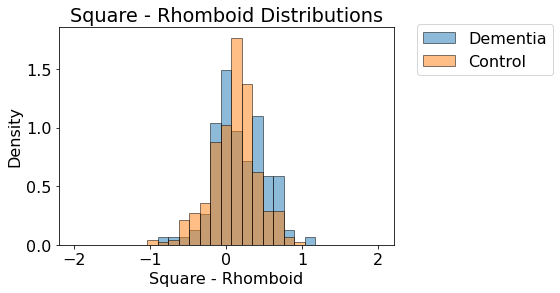

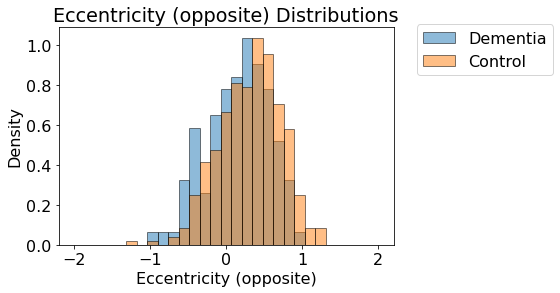


G H


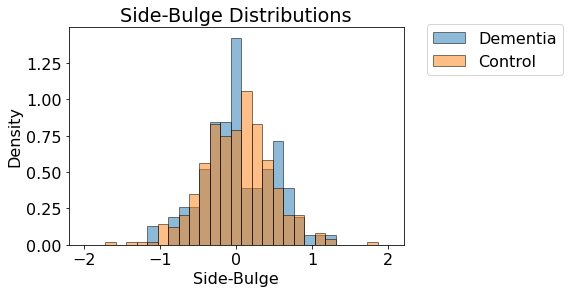

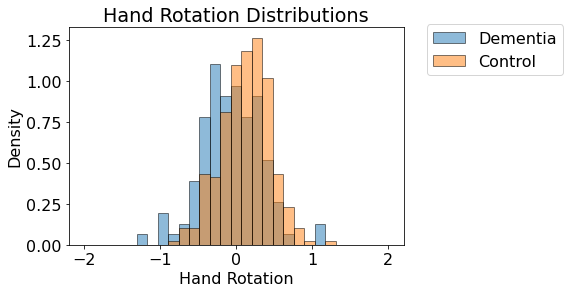


I J

**Supplementary Fig S2. Distributions of latent variables over dementia and controls in classification dataset**. **A.** Size, **B.** Obovate (avocado-ness), **C.** Oblate, **D.** Vertical displacement of clock hand assembly from center, **E.** Eccentricity, **F.** Angle between clock hands, **G.** Square-Rhomboid, **H.** Eccentricity (opp.), **I.** Side-bulge, **J.** Rotation of clock hand assembly.


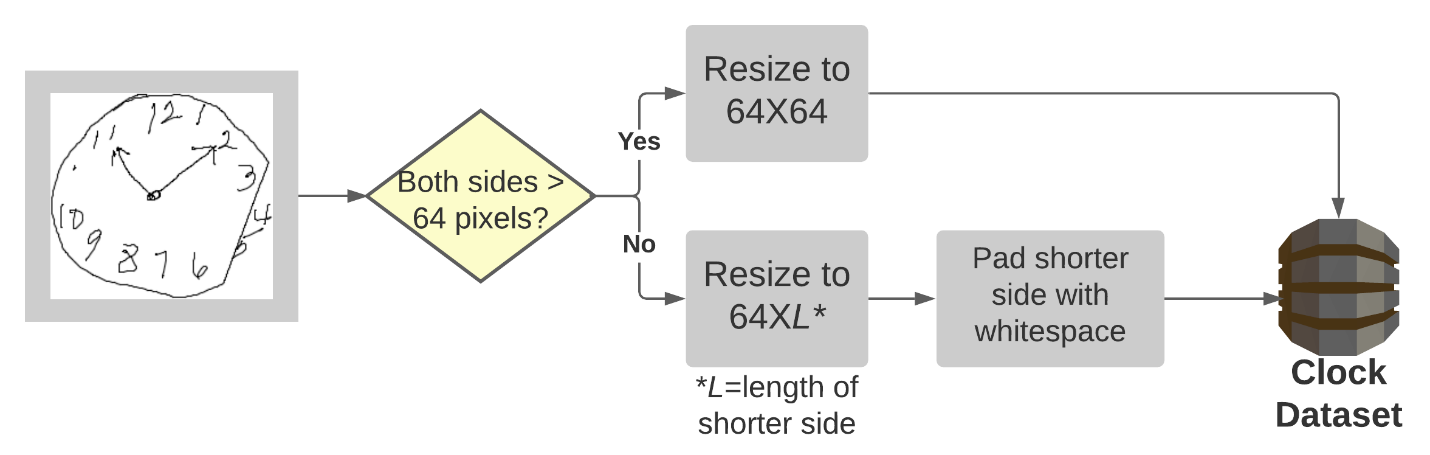


**Supplementary Fig S3. Preprocessing pipeline for clock drawings.** Resizing clock drawings to 64X64 might involve white-space padding if one side is initially smaller than 64 pixels.

**Supplementary Table 1. P-values after different corrections for Fig 3A.**

|  | Size | Obovate | Oblate | Vertical displ. of hands | Ellipse | Angle Between Hands | Square-Rhomboid | Opposite ellipse | Side bulge | Rotation of Hand assembly |
| --- | --- | --- | --- | --- | --- | --- | --- | --- | --- | --- |
| Two-tailed unequal var. | 2.25E-10 | 1.09E-06 | 2.65E-09 | 0.0002 | 0.0018 | 1.50E-08 | 0.078 | 0.0005 | 0.668 | 8.33E-06 |
| After Propensity-matching | 7.03E-07 | 1.43E-05 | 2.88E-10 | 0.0006 | 0.0006 | 4.95E-08 | 0.221 | 0.0038 | 0.974 | 0.0001 |
| Propensity-matching followed by multiple comparisons correction (BH) | 2.34E-06 | 3.57E-05 | 2.87E-09 | 0.0009 | 0.0009 | 2.47E-07 | 0.245 | 0.0047 | 0.975 | 0.0002 |

Abbreviation. BH; Benjamini-Hochberg.
